# Maternal Involvement in Education, Bicultural Acceptance, and School Adjustment: An Autoregressive Cross-Lagged Modeling Study among Adolescents from Multicultural Families

**DOI:** 10.3390/bs14050368

**Published:** 2024-04-26

**Authors:** Yangmi Lim

**Affiliations:** Department of Home Economics Education, College of Education, Jeonju University, Jeonju 55069, Republic of Korea; ym68@jj.ac.kr; Tel.: +82-63-220-2338

**Keywords:** maternal involvement in education, bicultural acceptance, school adjustment, multicultural families, autoregressive cross-lagged modeling

## Abstract

This study examined the associations between maternal involvement in education and bicultural acceptance and school adjustment during the first year of middle school among adolescents from Korean multicultural families as well as the reciprocal relationships between bicultural acceptance and school adjustment during the three years of middle school. The present study used three-wave longitudinal data of 1185 dyads of adolescents (50.8% girls; mean age = 12.96 ± 0.35 years at the first wave) and their immigrant mothers (mean age = 43.54 ± 5.19 years at the first wave), who participated in the Multicultural Adolescents Panel Study. An autoregressive cross-lagged modeling analysis revealed that maternal involvement in education was significantly and positively associated with adolescents’ bicultural acceptance and school adjustment in the first year of middle school. Individual levels of bicultural acceptance and school adjustment among adolescents remained moderately stable over the three years. Whereas the positive effects of school adjustment on bicultural acceptance were significant over time, the effects of bicultural acceptance on school adjustment were not. Finally, this study highlights the roles of intervention programs (e.g., parent and multicultural education) in facilitating maternal involvement in education and school adjustment as well as in increasing bicultural acceptance among minority youths.

## 1. Introduction

Since the late 1990s, South Korea has transformed into a multicultural society. This change is attributable to an increase in the number of international marriages as local governments have been driving cross-border marriages between rural bachelors and foreign brides. There has also been an influx of foreign residents in the aftermath of globalization and the Korean Wave [1]. Accordingly, the term “multicultural family”, a family form containing members from different cultural backgrounds, has emerged in South Korea. This family type applies primarily to families formed through international marriages [2]. In South Korea, international marriages exceeded 10% of the total number of marriages in 2010, accounting for 9.1% as of 2022 [3]. In 2022, most such unions (80.3%) involved marriages between Korean men and women from East or Southeast Asia, such as China, Vietnam, Japan, and the Philippines [4]. Therefore, multicultural families, formed through marriages between immigrant women and Korean men, have received increased policy attention [2].

Along with the growing number of long-term resident international marriages, the proportion of children from multicultural families in schools has also increased. South Korea has a single-track 6-3-3-4 education system, which denotes six years of elementary school, three years of middle school, three years of high school, and four years of university. As of 2023, there were 181,178 students from multicultural families in elementary, middle, and high schools––an increase of more than 3.9 times compared to 2012, which is in contrast to the 14% decrease in the total number of students during the same period due to low birth rates [5]. In many Western and non-Western societies, adolescence begins with entrance into middle school, during which adolescents must adapt to a school environment that is different from elementary school; they also undergo physiological and psychosocial changes that occur during puberty [6]. Furthermore, middle school students experience more pressure to obtain higher school grades and suffer more social pressures compared to elementary school students; they may also experience transformations in their relationships with parents and peers. Specifically, many parents become more involved in their children’s academic performance and interpersonal relationships, and peer relationships become more important in the children’s lives [7,8]. School adjustment relates to the ability of students to adapt to interacting with other people and comply with school classes and rules [8]. With the confluence of these developmental and contextual changes during this period, many adolescents experience difficulties in academic performance and psychosocial maladjustment at school [9,10].

Moreover, adolescents from multicultural families are more likely to be exposed to risk factors that impede school adjustment than those from non-multicultural families. Most adolescents from multicultural families who are enrolled in schools (80.6%) are children of international marriages born in South Korea [5]. They often experience identity confusion caused by differences in their parents’ cultural backgrounds, communication problems with foreign parents, and peer bullying due to skin color and appearance, all of which lead to psychosocial maladjustment [11]. Adolescents from multicultural families experience more school maladjustment and exhibit more depressive symptoms than children from non-multicultural families [12,13]. Furthermore, the school dropout rate for students from multicultural families was reported to be higher than that of students from non-multicultural families [14]; such students’ reasons for dropping out included bullying by peers and a lack of interest in academics as well as parental support [15]. However, several research findings [16] have indicated that there are no significant differences in school adjustment between youths from multicultural and non-multicultural families; thus, there is a need to systematically explore the variables that affect school adjustment among adolescents from multicultural families.

### 1.1. Maternal Involvement in Education and Adolescents’ School Adjustment and Bicultural Acceptance during the First Year of Middle School among Adolescents from Multicultural Families 

Various theories, research studies, and policies have identified the significant role of parental involvement in education for facilitating school adjustment and promoting academic achievement across elementary and secondary school levels [17]. Parental involvement in education and supportive parenting may play a pivotal role in students’ adaptation to school during the first year of middle school when the educational environment changes significantly and academic pressure increases [17,18]. According to Bronfenbrenner’s ecological systems theory [19], parents and schools are microsystems that directly influence children’s development and behaviors. Moreover, this theory posits that interconnections between two or more microsystems, called mesosystems, influence children’s development. Parental involvement in education is a mesosystem that links the home and school microsystems. Furthermore, an exosystem encompasses the broader social and environmental contexts that indirectly impact an individual’s development by affecting one of their inhabited microsystems, such as community resources and government policies. A macrosystem is a socio-cultural context (i.e., cultural beliefs, values, customs, and social norms) that influences the aforementioned environmental systems and human development [19,20].

Parental involvement in education refers to a set of specific activities that parents conduct to support their children’s learning and positive adjustment in schools. Parental involvement in education is divided into three categories: school-based involvement, home-based involvement, and home–school communication [21]. School-based involvement represents activities typically undertaken by parents in school settings for their children’s benefit (e.g., voluntary participation in school events). Home-based involvement refers to activities that parents engage in outside school to support their children’s education and schoolwork (e.g., help with homework). Home–school communication occurs when parents and school staff interact (e.g., talking with a school principal). Furthermore, several researchers [20,22] have defined parental involvement in education as activities performed by parents in relation to their children’s education, both at school and at home; these put the home–school communication factor into the category of school-based involvement.

Despite the consensus regarding the significance of parental involvement in education across developmental stages [23,24], research has produced conflicting findings regarding the effects of parental involvement in children’s education during middle school. Some research findings have indicated that parental involvement in education declines in terms of amount or effectiveness during middle school because adolescents may perceive such parental involvement in schools as interference with their autonomy [25]. In contrast, parental involvement in education was found to be positively associated with student achievement, school adjustment, and social relationships in middle school in previous studies [24,26,27].

Adolescents from multicultural families may require more school adjustment-related parental support than those from non-multicultural families because of their cultural backgrounds. However, the school participation of foreign parents has been reported to be limited [28]. In South Korea, where societal expectations require mothers to assume primary responsibility for raising children because of persistent gender role stereotypes [29], foreign mothers interact with their children more than fathers and take primary responsibility for providing support for their children’s school life and education [15]. In particular, although foreign mothers in multicultural families have the same level of interest in their children’s education as mothers from mainstream cultural backgrounds, they are unable to properly guide their children and participate in school education because of their lack of information about the Korean education system and their limited language competencies [30,31]. Foreign mothers in multicultural families have been reported to compensate for their lack of participation in school activities through home-based involvement and private tutoring support [32,33]. 

In South Korea, most pupils often receive private supplementary tutoring. Such tutoring can be received individually or in groups and sometimes in large classes. The tutoring content may be linked to specific lessons covered at school or encompass additional material [34]. In particular, some parts of East Asia (e.g., Japan and South Korea) are influenced by Confucian cultural traditions, which value education and effort in social success and achievement; thus, private tutoring has become an educational means for students to gain greater advantages for admission to prestigious universities by enhancing their test scores [34]. Despite several concerns about the negative impact of private tutoring (e.g., the household economic burden imposed by tutoring fees and the damage students’ preference for private tutoring causes to the capacity of public schooling) and some research results showing that the effectiveness of private education is limited [35], private tutoring has emerged as an alternative for supporting the education of children in multicultural families because foreign mothers from these families often lack guidance with regard to their children’s education [33]. Previous research findings [28,31] have indicated that higher levels of private tutoring and home-based educational support in multicultural families are consistently associated with higher levels of participation in learning activities and academic achievement among adolescents. Furthermore, prior studies [26,27] have reported that the higher the level of school-based involvement and home–school communication among foreign mothers from multicultural families, the higher the academic achievement and school adjustment of their adolescent children.

Maternal involvement in education also positively influences the development of bicultural acceptance among adolescents from multicultural families. Minority adolescents (including first-, second-, and third-generation immigrants) face the developmental task of forming and concretizing their own ethnic identities [36,37]. Succeeding in this task is often considered an important step for accomplishing the generic developmental tasks faced by youths [38]. According to research findings, immigrant youths’ bicultural acceptance of themselves was found to provide an optimal basis for successful development in adolescence and young adulthood [38,39]. Bicultural acceptance (or biculturalism) is an integrated type of acculturation, mainly experienced by the children of immigrant families. According to Berry’s model [40], one of the most well-known acculturation models, the acculturation types of family members with immigrant backgrounds are categorized into integration, assimilation, separation, and marginalization. Integration, known as bicultural acceptance, represents accepting the identity provided by both the heritage culture and the mainstream culture without resistance. While assimilation signifies adopting the mainstream culture and discarding the heritage culture, separation represents rejecting the mainstream culture and retaining the heritage culture. Marginalization refers to rejecting both the heritage and mainstream cultures.

School is a major context where mainstream cultural norms and values are introduced and reinforced, in which acculturation processes occur in minority youths. Parents are crucial agents of ethnic socialization among minority youths, influencing their children’s attitudes toward the heritage culture and other ethnic groups [41]. Therefore, when migrant parents actively participate in school activities and provide support for their children’s school adjustment, the children are more likely to show favorable attitudes toward the dominant culture [27,42]. Furthermore, higher levels of support from parents in multicultural families were associated with their children’s positive attitudes toward their parents’ attributes and cultural backgrounds [27,43].

### 1.2. Reciprocal Relationships between School Adjustment and Bicultural Acceptance during Middle School 

The results of extant research indicate that bicultural acceptance may have a reciprocal relationship with school adjustment among adolescents from multicultural families during middle school. Many studies [38,44,45,46] have reported that bicultural acceptance positively influences school adjustment among adolescents from multicultural families. Bicultural individuals are likely to be well-adjusted because they are competent in navigating both their dominant and heritage cultures and have social support networks from both cultures [47]. The relationship is not necessarily simply unidirectional. Several studies [27,48,49] have verified the effect of school life on bicultural acceptance by indicating that higher school adjustment predicts higher bicultural acceptance among adolescents from multicultural families. 

Some prior studies [45,48,49] have investigated the longitudinal relationship between school adjustment and bicultural acceptance, but they produced mixed results on the direction of effects between the two variables (bicultural acceptance → school adjustment or school adjustment → bicultural acceptance). In addition, Kim and Park [50] investigated the reciprocal causal relationships between multicultural acceptance and school adjustment among Korean middle school students but did not explore the relationship between bicultural acceptance and school adjustment by focusing on children from multicultural families. In particular, several longitudinal studies [50,51] have also reported that rank-order stabilities—which refer to the degree to which the relative ordering of individuals in a specific trait is maintained over time—in school adjustment, multicultural acceptance, and cultural identity are high. School adjustment and the formation of cultural identities are both major developmental tasks for middle school students from multicultural families. Thus, it is necessary to establish an intervention strategy that considers the degree of change in the two variables and the reciprocal causal relationships between the two variables over time to increase the effectiveness of intervention programs for minority youths. 

### 1.3. Aims and Hypotheses of This Study

This study aims to explore the associations between maternal involvement in education, school adjustment, and bicultural acceptance in the first year of middle school among adolescents from multicultural families and the reciprocal relationships between school adjustment and bicultural acceptance during the middle school period. Although previous studies [26,27,28,31] have identified the effect of maternal involvement in education on their children’s school adjustment and bicultural acceptance in multicultural families, none has simultaneously investigated the bidirectional effect between school adjustment and bicultural acceptance in the same study.

Based on the findings of previous research, the present study formulated the following hypotheses:

**Hypothesis** **1.** *Maternal involvement in education is positively associated with bicultural acceptance and school adjustment during the first year of middle school among adolescents from multicultural families.*

**Hypothesis** **2.** *Individual levels of bicultural acceptance and school adjustment remain stable during the three years of middle school among adolescents from multicultural families*.

**Hypothesis** **3.** *Bicultural acceptance and school adjustment are reciprocally and positively related to each other during the three years of middle school among adolescents from multicultural families*.

## 2. Materials and Methods

### 2.1. Participants and Procedure

This study utilized data from the Multicultural Adolescents Panel Study (MAPS) conducted by the National Youth Policy Institute (NYPI). The participants were children (primarily those born in Korea) and their foreign mothers from Korean multicultural families. When MAPS began in 2011, the sampling unit were schools. The sampling procedure was carried out in two stages. Sample schools were first selected using randomized stratified sampling based on the distribution of schools, including multicultural students in 16 cities and provinces across South Korea. Next, probability proportional sampling, which helps to increase the sampling rate of the schools with many multicultural students, was applied. The final sample was determined to be 1600 fourth-grade students (1600 mothers) drawn from 1000 elementary schools, which corresponds to 35.9% of the population of fourth-grade elementary school students from multicultural families (4452 students) in 2011. The sampling error, represented by the margin of error, was reported to be ±2.5% at the 95% confidence level. Therefore, there were no problems with the reliability of the survey and the representativeness of the population [52]. Since 2011, MAPS has conducted annual follow-ups with the participants. MAPS data from 2011 to 2019 are available to the public with permission from the NYPI. This study analyzed data measured from 2014 to 2016, when the participating children attended middle school. Finally, 1185 middle school students (583 boys, 602 girls; mean age in 2014: *M* = 12.96, *SD* = 0.35) and their foreign mothers (mean age in 2014: *M* = 43.54, *SD* = 5.19) from multicultural families, who had continuously participated in the three-wave survey and responded to all study variables, were selected as the final research participants.

Regarding these participants’ background characteristics, the most common country of origin for the mothers was Japan (37.2%), followed by the Philippines (25.7%) and ethnic Koreans in China (18.6%). Furthermore, the mothers were mostly high school graduates (46.5%), second- or third-year college graduates (26.4%), and university graduates (15.4%). The majority of the mothers regarded themselves as having high levels of Korean language proficiency (66.7%), whereas almost all of the adolescents perceived themselves as proficient in the Korean language (97.5%). However, 71.6% of the adolescents evaluated themselves as having low levels of proficiency in their foreign mothers’ native languages.

### 2.2. Measures

#### 2.2.1. Maternal Involvement in Education

The participating mothers’ self-reported levels of maternal involvement in education were measured using the home- and school-based involvement in education scales developed by the MAPS research team [53]. The home-based involvement in education scale comprises eight items measuring the degree of maternal involvement in education and efforts expended at home to support children’s school adjustment and improve their academic performance (e.g., “I provide private tutoring to help my children improve their grades”). This scale originally consisted of nine items, but one of the items (“I consult a school teacher about my children”) was removed because it overlapped with an item included in the school-based involvement in education scale in terms of its contents. Each item is rated on a five-point scale, ranging from not at all (1) to very much so (5). The possible score range was 8–40, with higher scores indicating a greater degree of the mothers’ home-based involvement in the students’ education.

The school-based involvement in education scale consists of nine items assessing the degree of the parents’ participation in education-related activities (e.g., parent meetings) at school and communication with school staff, such as teachers, about their children’s school life. Each item is answered on a four-point scale (never = 1 to four or more times per year = 4), with higher scores representing a greater level of maternal school-based involvement (score range: 9–36). This study used home- and school-based involvement in education as the indices of maternal involvement in education in an autoregressive cross-lagged (ARCL) modeling analysis. The internal consistency coefficient (Cronbach’s α) of the home-based involvement in education scale was 0.72; the Cronbach’s α was 0.86 for the school-based involvement in education scale.

#### 2.2.2. School Adjustment

The scale developed by Kim et al. [54] was used to measure the adolescents’ perceived school adjustment. This scale was divided into three subscales, each comprising five items: learning activities (e.g., “I always do my school homework”), peer relationships (e.g., “When I fight with a friend, I apologize first”), and relationships with teachers (e.g., “When I meet teachers, I greet them warmly”). Each item was answered on a four-point scale (not at all = 1 to very much so = 4), with higher scores indicating a higher level of school adjustment (score range: 5–20). This study employed learning activities, peer relationships, and relationships with teachers as the indices of school adjustment in the ARCL modeling analysis. The Cronbach’s α values measured over the three years of middle school for the learning activities subscale were 0.77 (first grade), 0.78 (second grade), and 0.78 (third grade); 0.60 (first grade), 0.62 (second grade), and 0.63 (third grade) for the peer relationships subscale; and 0.89 (first grade), 0.88 (second grade), and 0.89 (third grade) for the relationships with teachers subscale.

#### 2.2.3. Bicultural Acceptance

The level of bicultural acceptance perceived by the adolescents was measured using a scale revised by the MAPS research team [53] based on Nho and Hong’s scale [39]. This scale consists of 10 items measuring the degree of the adolescents’ acceptance of Korean culture and their mothers’ native culture (e.g., “It is important for me to learn Korean culture (or the culture of my foreign parent’s country of origin)”). Each item was rated on a four-point scale (not at all = 1 to very much so = 4), with higher scores representing a greater level of bicultural acceptance toward both South Korea and the foreign mother’s country of origin (score range: 10–40).

This study used two observed variables (two parcels), calculated using the random item parceling method employed by several scholars [55], as bicultural acceptance indices in the ARCL modeling analysis. Utilizing this item parceling technique helps to ensure the normality of the data while reducing random errors [55]. The Cronbach’s α values of the bicultural acceptance scale measured during the three years of middle school were 0.87 (first grade), 0.87 (second grade), and 0.87 (third grade).

### 2.3. Statistical Analysis

This study conducted analyses of the relevant data using IBM SPSS Statistics (version 26.0) and AMOS 26.0. First, the descriptive statistics of the participants’ demographic characteristics and the general tendencies of the measured variables were calculated. Moreover, Pearson correlation coefficients measuring the correlation values between the two research variables were calculated.

To examine the effects of maternal involvement in education on the adolescents’ school adjustment and bicultural acceptance during the first year of middle school, the cross-time stabilities of bicultural acceptance and school adjustment, as well as the bidirectional longitudinal relationships between school adjustment and bicultural acceptance during the three years of middle school, an ARCL modeling analysis was conducted. ARCL modeling analysis can extract autoregressive and cross-lagged effects in the amount of change across time in two or more constructs. The autoregressive effect represents the effect of a given construct on itself when measured at a later time. In contrast, the cross-lagged effect refers to the effect of a construct on another construct measured on a later occasion. ARCL modeling analysis is performed by sequentially testing the invariances of measurement, path, and error covariance in that order. A measurement invariance test is conducted to demonstrate whether the same construct was measured at each time point in a longitudinal study. Path invariance verifies whether the autoregressive coefficients of the latent variables and the cross-lagged coefficients between latent variables are stable over time. Finally, the invariance of error covariances is determined by verifying whether the covariances of residual errors are stable over time [56,57].

**Figure 1:**
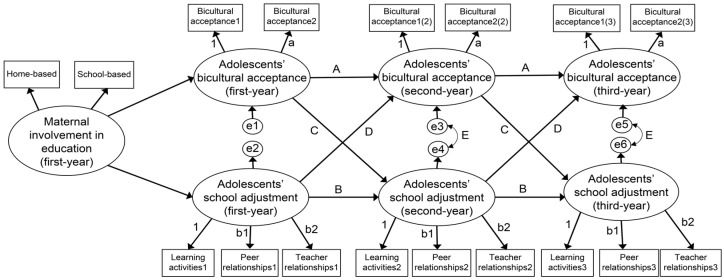
Research model. Note. a = equality constraints on all factor loadings of bicultural acceptance; b1, b2 = equality constraints on all factor loadings of school adjustment; A = constraints on all autoregressive coefficients of bicultural acceptance; B = constraints on all autoregressive coefficients of school adjustment; C = equality constraints on all cross-lagged coefficients of bicultural acceptance on school adjustment; D = equality constraints on all cross-lagged coefficients of school adjustment on bicultural acceptance; E = equality constraints on all covariances of residual errors in bicultural acceptance and school adjustment.

This research model is presented in Figure 1, wherein equality constraints imposed on measurement, path, and error covariance are included. Model fitness was evaluated using goodness-of-fit criteria. Of the model fit indices, the chi-squared value is highly sensitive to the sample size; if the sample size is greater than 300, *p* tends to be less than 0.05. The current sample size was 1185; thus, the indices that were not sensitive to the sample size were used: comparative fit index (CFI), normed fit index (NFI), and root-mean-squared error of approximation (RMSEA). CFI and NFI values of 0.90 or higher and an RMSEA value of 0.08 or lower indicated a good fit [58]. The invariance tests of measurement, path, and error covariance were performed by comparing the goodness-of-fit indices between the models with added constraints and the previous models sequentially. Specifically, if the values of the goodness-of-fit indices of the model with added constraints (CFI, NFI, and RMSEA) were better than or equal to those of the previous model or did not decrease by more than 0.01 compared to those of the previous model, equivalence was considered to be established between the two models [59].

## 3. Results

### 3.1. Descriptive Statistics and Correlations

Table 1 shows the mean values and standard deviations of the observed variables and their correlations. First, the mean values of the items pertaining to maternal home-based (five-point scale) and school-based (four-point scale) involvement in education measured in the first year of middle school were 2.42 (*SD* = 0.57) and 1.42 (*SD* = 0.41), respectively. Considering the possible scoring range of each scale, the mean values of maternal home- and school-based involvement in education were all lower than the midpoint scores. However, the adolescents’ average scores for bicultural acceptance during the three waves (from the first to the third year of middle school) were relatively high—that is, from 2.90 (*SD* = 0.38) to 2.94 (*SD* = 0.39)—out of a possible score of four. The mean values of the adolescents’ school adjustment subscales (four-point scale) during the three waves (from the first to the third year of middle school) were relatively high, with learning activities having scores from 2.87 (*SD* = 0.52) to 2.88 (*SD* = 0.52); peer relationships, from 3.17 (*SD* = 0.41) to 3.18 (*SD_second-year_* = 0.39, *SD_third-year_* = 0.37); and relationships with teachers, from 3.08 (*SD* = 0.56) to 3.17 (*SD* = 0.56). Skewness and kurtosis were also examined to determine whether the variables satisfied the assumptions of multivariate normality in the ARCL modeling analysis. The variables satisfied the threshold suggested by West et al. [60] (absolute skewness value < 2 and absolute kurtosis value < 7 indicate normality). The correlational analysis (Table 1) showed that maternal home- and school-based involvement in education measured during the first year of middle school were both significantly and positively correlated with bicultural acceptance and all school adjustment indices (learning activities, peer relationships, and relationships with teachers). The bicultural acceptance and all school adjustment indices measured earlier (first or second year of middle school) were significantly and positively correlated with the same variables measured later (second or third year of middle school). Furthermore, bicultural acceptance at an earlier stage (first or second year of middle school) was significantly and positively correlated with all school adjustment indices (learning activities, peer relationships, and relationships with teachers) at a later stage (second or third year of middle school). Finally, each of the school adjustment indices (learning activities, peer relationships, and relationships with teachers) at an earlier stage (first or second year of middle school) was significantly and positively correlated with bicultural acceptance at a later stage (second or third year of middle school).

### 3.2. Longitudinal Relationships among Maternal Involvement in Education, Adolescents’ School Adjustment, and Bicultural Acceptance

An ARCL modeling analysis was performed to examine the relationships between maternal involvement in education and adolescents’ bicultural acceptance and school adjustment in the first year of middle school, the cross-time stabilities of bicultural acceptance and school adjustment, and the reciprocal causal relationships between the two variables during the three years of middle school. To determine a final model, the eight competitive models were sequentially developed to verify measurement, path, and error covariance invariances. Goodness-of-fit indices were calculated for each of the eight models to determine a final model (Table 2). As each model had a nested relationship with the subsequent model with added constraints, the final model was determined by sequentially comparing the goodness-of-fit indices (i.e., CFI, NFI, and RMSEA) corresponding to the constraint-added and previous models.

First, when equality constraints for all factor loadings of bicultural acceptance and school adjustment were added in Models 2 and 3, the values of CFI, NFI, and RMSEA did not worsen by more than 0.01 from Model 1 to 2 and from Model 2 to 3. Therefore, the measurement invariances of bicultural acceptance and school adjustment were all supported. Next, when equality constraints were imposed on the autoregressive coefficients of bicultural acceptance and school adjustment in Models 4 and 5, the values of CFI, NFI, and RMSEA did not worsen by more than 0.01 both from Model 3 to 4 and from Model 4 to 5. Thus, the invariances of autoregressive coefficients of bicultural acceptance and school adjustment were all verified. Furthermore, there were no differences between Models 5 and 6 or between Models 6 and 7, as the CFI and NFI values of Models 6 and 7 did not decrease by more than 0.01 compared to the corresponding goodness-of-fit index values of each previous model, and the values of RMSEA did not change from Model 5 to 7. Therefore, the invariances of all cross-lagged effects of bicultural acceptance (or school adjustment) at an earlier stage on school adjustment (or bicultural acceptance) at a later stage were also confirmed. Moreover, the values of CFI and NFI did not change, and the RMSEA improved from Model 7 to 8; as such, all error covariances between bicultural acceptance and school adjustment were equal over time. Consequently, Model 8, in which all the invariances of the measurement, path, and error covariance were verified over time, was selected as the final model for this study.

**Figure 2:**
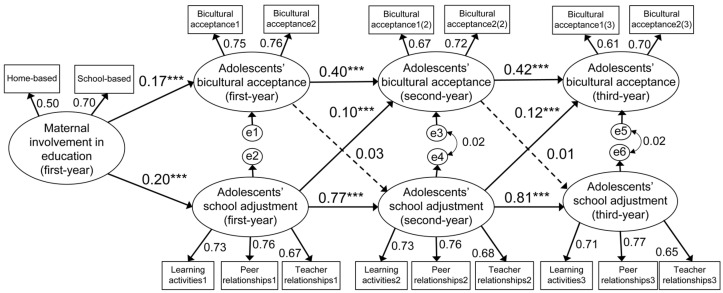
Longitudinal relationships among maternal involvement in education, adolescents’ school adjustment, and bicultural acceptance (*N* = 1185). Note: The numbers shown above the paths are standardized regression coefficients. *** *p* < 0.001.

The standardized path coefficients of the final selected model are shown in Figure 2. Both the CFI (0.907) and RMSEA (0.077) values of the final model satisfied the threshold criteria (CFI ≥ 0.90, RMSEA ≤ 0.08), and the NFI value (0.896) also almost reached the threshold of 0.90; thus, the fit of the final model was overall adequate. Maternal involvement in education was significantly and positively associated with adolescents’ bicultural acceptance and school adjustment during the first year of middle school. Additionally, adolescents’ bicultural acceptance and school adjustment at earlier time points had significant and positive effects on the same variables at later time points during the three years of middle school; in particular, given that the autoregressive coefficients of bicultural acceptance and school adjustment lay between 0.40 and 0.81, overall, the adolescents’ levels of bicultural acceptance and school adjustment remained moderately stable over time. However, the adolescents’ bicultural acceptance, measured at an earlier stage, did not exert any effect on their school adjustment, as measured at a later stage. In contrast, the adolescents’ school adjustment, measured at an earlier stage, had significant and positive effects on their bicultural acceptance, which was measured at a later stage.

## 4. Discussion

This study examined the relationships between maternal involvement in education, bicultural acceptance, and school adjustment in the first year of middle school and the bidirectional effects between bicultural acceptance and school adjustment during the three years of middle school among adolescents from multicultural families.

First, the results of this study revealed that maternal involvement in education was positively related to the adolescents’ bicultural acceptance and school adjustment during their first year of middle school. Therefore, Hypothesis 1 was supported. This result is congruent with the findings of earlier studies indicating that maternal home- and school-based involvement in education has positive influences on their adolescent children’s school adjustment [26,27,28,31] and bicultural acceptance [27,42,43] in multicultural families. In particular, this study comprehensively analyzed maternal school- and home-based involvement. The results of this study show that the strength of the relationships between maternal involvement in education and school adjustment and bicultural acceptance was stronger compared to the results of the study by Lim [27], wherein the MAPS data measured during the first year of middle school, like in this study, was used, but only maternal school-based involvement in education was analyzed. Parental involvement in education at home and school can be regarded as a form of social capital that supports students’ academic motivation and school adjustment; specifically, parental involvement in education promotes norms of reciprocity (a social norm where, if someone does something beneficial for you, you must return the favor) in their children by conveying to them the importance of education. This, in turn, might lead to a greater academic achievement and more responsible behaviors among children in schools. In addition, parental involvement increases their social connections with school teachers, thereby encouraging positive interactions with them [61]. Consequently, such parents’ children feel satisfied with school life and are able to adapt well.

Furthermore, the current study results concerning the positive relationship between maternal involvement in education and children’s bicultural acceptance may be explained by the mother’s acculturation type. Prior research findings found that foreign mothers with an integration acculturation type showed the most positive parenting behaviors toward their children [62]. Therefore, as mothers who actively support their children’s school adjustment and academic work are more likely to have an integration acculturation (bicultural acceptance) type, their children’s bicultural acceptance may be strengthened through direct teaching from the mothers and the children’s observations of their mothers’ behaviors.

Second, this study also found that the levels of bicultural acceptance and school adjustment among adolescents from multicultural families were moderately stable over the three waves; specifically, the higher the level of bicultural acceptance and school adjustment at an earlier stage, the higher the level of each of the same variables at a later stage. Thus, Hypothesis 2 was supported. This finding is in line with previous results that have reported on the cross-time stabilities of individual differences in bicultural acceptance and school adjustment during adolescence [50,51].

Moreover, after controlling for the autoregressive effects of school adjustment and bicultural acceptance, the positive effects of school adjustment on bicultural acceptance were significant during the middle school period. However, the effects of bicultural acceptance on school adjustment were not supported in this study. Therefore, Hypothesis 3 was partially supported. This finding is compatible with previous longitudinal research [48,49,50] showing a positive effect of school adjustment on bicultural acceptance. Comprehensively, the present study supports an adjustment → biculturalism effect rather than a biculturalism → adjustment effect.

Adolescence is a period during which one builds one’s identity while interacting with one’s environment; especially for minority adolescents, ethnicity can play an important role in their identity development [63]. School is a key acculturation context for youths with diverse migrant backgrounds (e.g., refugees and first- and second-generation immigrants). Relationships with teachers and peers at school can affect ethnic identity development through the school connectedness developed by youths from multicultural families [64]. School connectedness refers to the students’ attachment, affiliation, and bonding with the school’s environment and personnel [65,66]. For minority youths, feeling connected and accepted in a school environment can potentially provide a sense of belonging, leading to more positive emotions, positive appraisals of the dominant culture, and potentially better acculturation [40,67]. Furthermore, when minority youths attain academic achievements in schools where a dominant cultural orientation is prevalent, they may gain sufficient time and resources to explore and reconnect with their heritage culture. Consequently, these well-adjusted individuals are likely to be highly oriented to both cultures (i.e., bicultural) [47].

In sum, unlike the longitudinal studies that have focused on the one-way effect of bicultural acceptance on school adjustment, or vice-versa, this study contributes to the literature by identifying the effect of school adjustment on bicultural acceptance among middle school students from multicultural families by applying a bidirectional model of the two variables. It is also meaningful that, in this process, the reliability of the research results was improved by conducting an autoregressive cross-lagged modeling analysis that allowed for the identification of the bidirectional effects between two variables while controlling the autoregressive effects of school adjustment and bicultural acceptance.

This study’s findings have practical implications for schools and local communities. First, it is imperative to increase the level of maternal school-based involvement in the education of first-grade children in middle school to facilitate school adjustment and bicultural acceptance among the children. Foreign mothers in multicultural families generally have a low level of participation in school-based activities owing to their lack of information about Korean education and limited Korean language skills [28]; therefore, they tend to compensate for their low level of school-based involvement through private tutoring support [32,33]. Furthermore, immigrant mothers’ involvement in schools could reduce their degree of parenting efficacy and support for children because of the territorial behaviors of teachers and mothers from dominant cultural backgrounds and their prejudices against multicultural families [68]. Consistent with the results of these previous studies, the descriptive statistics of this study showed that the average score for school-based involvement in education (1.42 ± 0.41, four-point scale) was lower than that for home-based involvement in education (2.42 ± 0.57, five-point scale) among the participating mothers. Therefore, to encourage immigrant mothers to participate in school-based activities and programs, schools must provide information on school education and child education counseling services by running parent education and parent-mentoring programs (using parent mentors with experience in raising adolescent children) in connection with local community organizations.

Moreover, at the national level, various services, including parent education, should be provided to aid the school adjustment of children from multicultural families. Currently, South Korea operates the “Customized Integrated Support Project” for students from multicultural families experiencing difficulties in schools (e.g., peer bullying and academic problems); it has brought in experts from relevant fields to provide guidance that is tailored to the problems of students since 2022 [69]. This project will continue to address the issue of cultural identity along with school adjustment regarding children from multicultural families. It should also implement a parent education program containing the information and education necessary for parents to provide their children with adequate support at home.

In addition, Korean society has stood on the principle of “Danil Minjok”, a singular ethnic group connected by one blood, one language, and one culture [70]; as such, native-born individuals with such prejudices may have negative feelings toward multicultural families and act violently toward them because they perceive them as “destroying” the ethnic homogeneity of their own culture. Therefore, to increase multicultural acceptance among teachers, staff, and students in school settings, multicultural education—any form of education that fosters the ability and attitude to respect all human beings by breaking away from prejudice based on race, ethnicity, disability, and so on—should be actively carried out from early childhood. Teachers should also have the opportunity to strengthen their understanding of children from multicultural families and their teaching competencies through pre-service and in-service teacher education programs.

Lastly, this study provides directions for future research in light of its limitations. Unlike in previous studies [45,46], the longitudinal effect of bicultural acceptance on school adjustment was not supported in this study. Therefore, future studies should continue to examine the reciprocal relationships between school adjustment and bicultural acceptance over time from middle school to high school. Second, previous studies [71,72] have reported that fathers, apart from mothers, have an independent influence on their children’s bicultural acceptance and school adjustment; thus, future prospective research should compare the relative influences of parents on bicultural acceptance and school adaptation among adolescents from multicultural families. Third, as parents’ influence on their children’s school adjustment and cultural identities was found to differ according to the children’s sex in several studies [72,73], future research could compare the influence of parents’ involvement in education on adolescent children’s school adjustment and bicultural acceptance while considering the effect of the children’s sex.

## 5. Conclusions

Based on the three-wave longitudinal data drawn from a national survey, this study’s findings revealed that maternal involvement in education was significantly and positively related to adolescents’ bicultural acceptance and school adjustment during the first year of middle school. While adolescents’ school adjustment exerted positive effects on bicultural acceptance across the three years of middle school, the effects of bicultural acceptance on school adjustment were not supported during the same period. To encourage foreign mothers’ involvement in education, this study recommends that the government support schools in providing parent education programs and counseling programs to assist foreign parents in multicultural families with children in middle school; in this way, they can obtain information about school education and learn how to offer appropriate support to their children. Furthermore, this study highlights the role of multicultural education implemented from early childhood in increasing multicultural acceptance among peers, teachers, and staff at school and that of teacher education in helping children from multicultural families to adapt to school.

## Figures and Tables

**Table 1:** Descriptive statistics and correlations among the variables (*N* = 1185).

	1.1	1.2	2	3.1	3.2	3.3	4	5.1	5.2	5.3	6	7.1	7.2	7.3
1.1														
1.2	0.40 **													
2	0.08 **	0.10 **												
3.1	0.12 **	0.13 **	0.34 **											
3.2	0.06 *	0.09 **	0.36 **	0.56 **										
3.3	0.08 **	0.08 **	0.32 **	0.48 **	0.51 **									
4	0.03	0.11 **	0.44 **	0.25 **	0.27 **	0.18 **								
5.1	0.07 *	0.11 **	0.25 **	0.61 **	0.38 **	0.31 **	0.36 **							
5.2	0.07 *	0.07 *	0.26 **	0.39 **	0.51 **	0.50 **	0.41 **	0.51 **						
5.3	0.04	0.06 *	0.27 **	0.36 **	0.33 **	0.40 **	0.41 **	0.53 **	0.51 **					
6	0.03	0.05	0.40 **	0.23 **	0.23 **	0.18 **	0.49 **	0.23 **	0.32 **	0.28 **				
7.1	0.11 **	0.14 **	0.26 **	0.55 **	0.37 **	0.32 **	0.28 **	0.63 **	0.44 **	0.36 **	0.31 **			
7.2	0.08 **	0.05	0.23 **	0.35 **	0.43 **	0.27 **	0.34 **	0.36 **	0.55 **	0.36 **	0.40 **	0.53 **		
7.3	0.11 **	0.08 **	0.20 **	0.31 **	0.33 **	0.37 **	0.26 **	0.35 **	0.36 **	0.47 **	0.31 **	0.45 **	0.49 **	
*M*	2.42	1.42	2.92	2.88	3.17	3.17	2.90	2.87	3.18	3.08	2.94	2.88	3.18	3.11
*SD*	0.57	0.41	0.39	0.52	0.41	0.56	0.38	0.52	0.39	0.56	0.39	0.52	0.37	0.56
S	0.20	1.09	0.03	−0.08	0.02	−0.23	0.27	−0.01	0.05	−0.15	−0.07	−0.15	0.22	−0.29
K	0.03	0.90	0.80	0.16	0.04	0.34	0.43	0.03	0.24	0.19	0.95	0.34	0.25	0.51

Note: 1.1. Home-based involvement in education (first year); 1.2. School-based involvement in education (first year); 2. Bicultural acceptance (first year); 3.1. Learning activities (school adjustment, first year); 3.2. Peer relationships (school adjustment, first year); 3.3. Relationships with teachers (school adjustment, first year); 4. Bicultural acceptance (second year); 5.1. Learning activities (school adjustment, second year); 5.2. Peer relationships (school adjustment, second year); 5.3. Relationships with teachers (school adjustment, second year); 6. Bicultural acceptance (third year); 7.1. Learning activities (school adjustment, third year); 7.2. Peer relationships (school adjustment, third year); 7.3. Relationships with teachers (school adjustment, third year). *M* = mean; *SD* = standard deviation; S = skewness; K = kurtosis. * *p* < 0.05; ** *p* < 0.01.

**Table 2:** Test for the invariance of the autoregressive cross-lagged model (*N* = 1185).

Models	*χ*^2^(*df*)	CFI	NFI	RMSEA	Δ CFI	Δ NFI	Δ RMSEA
Model 1	788.891(104)	0.922	0.911	0.075			
Model 2	824.634(106)	0.918	0.907	0.076	−0.004	−0.004	0.001
Model 3	830.483(110)	0.918	0.906	0.074	0.000	−0.001	−0.002
Model 4	915.260(111)	0.909	0.897	0.078	−0.009	−0.009	0.004
Model 5	918.173(112)	0.909	0.897	0.078	0.000	0.000	0.000
Model 6	918.495(113)	0.908	0.897	0.078	−0.001	0.000	0.000
Model 7	925.729(114)	0.907	0.896	0.078	−0.001	−0.001	0.000
Model 8	927.075(115)	0.907	0.896	0.077	0.000	0.000	−0.001

Note: Model 1 = the base model in which equality constraints are not imposed; Model 2 = the model in which equality constraints are added for all factor loadings of bicultural acceptance; Model 3 = the model in which equality constraints are added for all factor loadings of school adjustment; Model 4 = the model in which equality constraints are added for all autoregressive coefficients of bicultural acceptance; Model 5 = the model in which equality constraints are added for all autoregressive coefficients of school adjustment; Model 6 = the model in which equality constraints are added for all cross-lagged coefficients of bicultural acceptance on school adjustment; Model 7 = the model in which equality constraints are added for all cross-lagged coefficients of school adjustment on bicultural acceptance; Model 8 = the model in which equality constraints are added for all covariances of residual errors in bicultural acceptance and school adjustment.

## Data Availability

This study was conducted using publicly available national data (https://www.nypi.re.kr/archive/mps/program/examinDataCode/view?menuId=MENU00226&pageNum=1&titleId=146&schType=0&schText=&firstCategory=&secondCategory=; accessed on 5 September 2023). The data can be accessed with permission from the National Youth Policy Institute.
